# Dilute Bicelles for Glycosyltransferase Studies, Novel Bicelles with Phosphatidylinositol

**DOI:** 10.1021/acs.jpcb.2c02327

**Published:** 2022-07-26

**Authors:** Joan Patrick, Mikel García Alija, Jobst Liebau, Pontus Pettersson, Ane Metola, Lena Mäler

**Affiliations:** Department of Biochemistry and Biophysics, Stockholm University, SE-106 91 Stockholm, Sweden

## Abstract

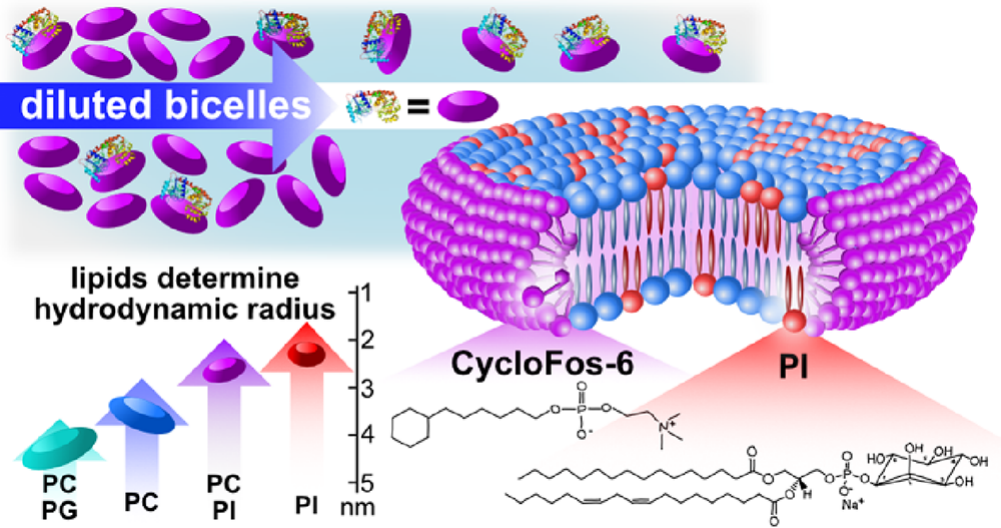

Solution-state NMR can be used to study protein–lipid interactions, in particular, the effect that proteins have on lipids. One drawback is that only small assemblies can be studied, and therefore, fast-tumbling bicelles are commonly used. Bicelles contain a lipid bilayer that is solubilized by detergents. A complication is that they are only stable at high concentrations, exceeding the CMC of the detergent. This issue has previously been addressed by introducing a detergent (Cyclosfos-6) with a substantially lower CMC. Here, we developed a set of bicelles using this detergent for studies of membrane-associated mycobacterial proteins, for example, PimA, a key enzyme for bacterial growth. To mimic the lipid composition of mycobacterial membranes, PI, PG, and PC lipids were used. Diffusion NMR was used to characterize the bicelles, and spin relaxation was used to measure the dynamic properties of the lipids. The results suggest that bicelles are formed, although they are smaller than “conventional” bicelles. Moreover, we studied the effect of MTSL-labeled PimA on bicelles containing PI and PC. The paramagnetic label was shown to have a shallow location in the bicelle, affecting the glycerol backbone of the lipids. We foresee that these bicelles will be useful for detailed studies of protein–lipid interactions.

## Introduction

Membrane homeostasis is controlled by the fine-tuned synthesis of lipids, and in bacteria, glycosyltransferases (GTs) play an important role in the control of the synthesis of glycosylated lipids.^1^ GTs involved in adding sugar moieties to lipid molecules are membrane-associated as they must bring together a membrane-embedded lipid acceptor substrate and a soluble sugar donor, implying that they are involved in phase transfer catalysis.^2,3^ As there are a large number of similar lipid substrates, GTs need to be highly specific: the interaction between membrane lipids and the enzyme needs to be well-tuned to match the characteristics of the substrate in order for binding to occur. For example, among bacterial membrane-associated enzymes of the GT-B fold, it has been demonstrated that the 1,2-diacylglyceroltransferase (MGS) from *Acholeplasma laidlawii* that transfers a glucose moiety to diacylglycerol, binds to lipid membranes more or less irreversibly and can best be described as a monotopic membrane protein.^4,5^ In contrast, WaaG in *Escherichia coli*, which is involved in lipopolysaccharide synthesis, has been shown to bind to lipids with a much lower affinity, indicating that this protein is instead best described as a peripheral membrane-associated enzyme.^6^ Similar observations have been made for PimA, a key enzyme for mycobacterial growth that initiates synthesis of lipid components in, e.g., *Mycobacterium tuberculosis*.^7^ These observations clearly demonstrate that GTs are finely tuned to match the properties of the lipid membranes. Moreover, these findings indicate that in order to understand the underlying properties by which these enzymes are activated, it is necessary to study membrane interactions on a molecular level.

Membrane interactions of membrane-associated proteins in general and of GTs in particular have been studied by a wide range of biophysical methods, including a range of spectroscopic techniques, such as circular dichroism (CD) and fluorescence spectroscopy.^8^ Solution-state NMR has been used among others to pinpoint the location of proteins in a lipid environment. For example, the structure induction by lipids, the location of the proteins in the bilayer and the effect of different lipids on binding has been studied for WaaG^8^ and MGS.^5^ For these purposes, small, fast-tumbling bicelles have been used. Such bicelles were first demonstrated to be of use for solution-state NMR studies of protein-lipid interactions in the 1990s,^9^ and since then, their morphology has been widely studied. Although other membrane mimetic media, such as nanodiscs,^10−12^ are argued to be better for structural studies of proteins, small isotropic bicelles have been particularly useful for studying lipid properties and their dependence on the presence of, e.g., proteins. Bicelles are, in this case, ideal since they allow the recording of high-resolution NMR spectra for ^1^H, ^13^C, and ^31^P in the lipids, which has been exploited for studying the rotational dynamics of lipids.^13^

Small bicelles are versatile mixtures of lipids and detergents, and at relatively high detergent concentrations that substantially exceed the detergent’s critical micelle concentration (CMC), they have been demonstrated by a number of methods to form fast-tumbling, disc-like particles.^14−17,13,18,19^ Bicelles have been developed specifically to mimic, e.g., the inner membrane in *E. coli* for studies of bacterial enzymes,^20,21^ or to mimic chloroplastic membranes for studies of plant glycosyltransferases.^22^ It has been shown by solution-state NMR that at high lipid-to-detergent ratios (called *q*-value), detergents and lipids tend to segregate into a detergent rim and a lipid bilayer core.^14,15,23^ It has been argued that at very low lipid-to-detergent ratios, the mixtures are better described as mixed micelles,^24^ while other studies have indicated that true bicelles are formed but that this is dependent on many factors, such as the concentration of both lipids and detergent. In order to obtain discoidal bicelles it is thus necessary to (1) have a “high” *q*-ratio and (2) to exceed the detergent CMC by roughly one order of magnitude.^18,25^

In consequence, one of the main problems with using bicelles is the need for a relatively high concentration of detergent, and thus, a corresponding high lipid concentration is also required. Moreover, in order to be amenable to solution NMR study, the concentration ratio of lipid to detergent (*q*-value) needs to be in a range that allows for relatively small bicelles to be formed (*q* < 1) while ensuring that enough lipid molecules are present to form bicelles, i.e., the *q*-value must not be too low. The commonly used detergent dihexanoylphosphatidylcholine (DHPC) has a CMC of ∼10 mM so that a bicelle mixture with a *q*-value of 0.5 requires at least 100 mM DHPC and 50 mM lipids. This can be problematic when the effect of peripheral membrane proteins on lipid properties is to be studied as sufficiently high protein concentrations cannot be achieved for the interaction to be observed. In this case, only a fraction of the lipids are in contact with a protein, and effects may thus be unobservable. Additionally, it is desirable to reduce the amount of lipids required for an experiment. Lu et al. addressed these problems by using 6-cyclohexyl-1-hexylphosphocholine (Cyclofos-6) as the detergent that has a CMC of around 3 mM.^26^ In this way, the overall concentration of lipids and detergents could be lowered. Care must be taken, however, to ensure that the lipid properties as well as the bilayer-like properties within the bicelles are retained.

**Figure 1:**
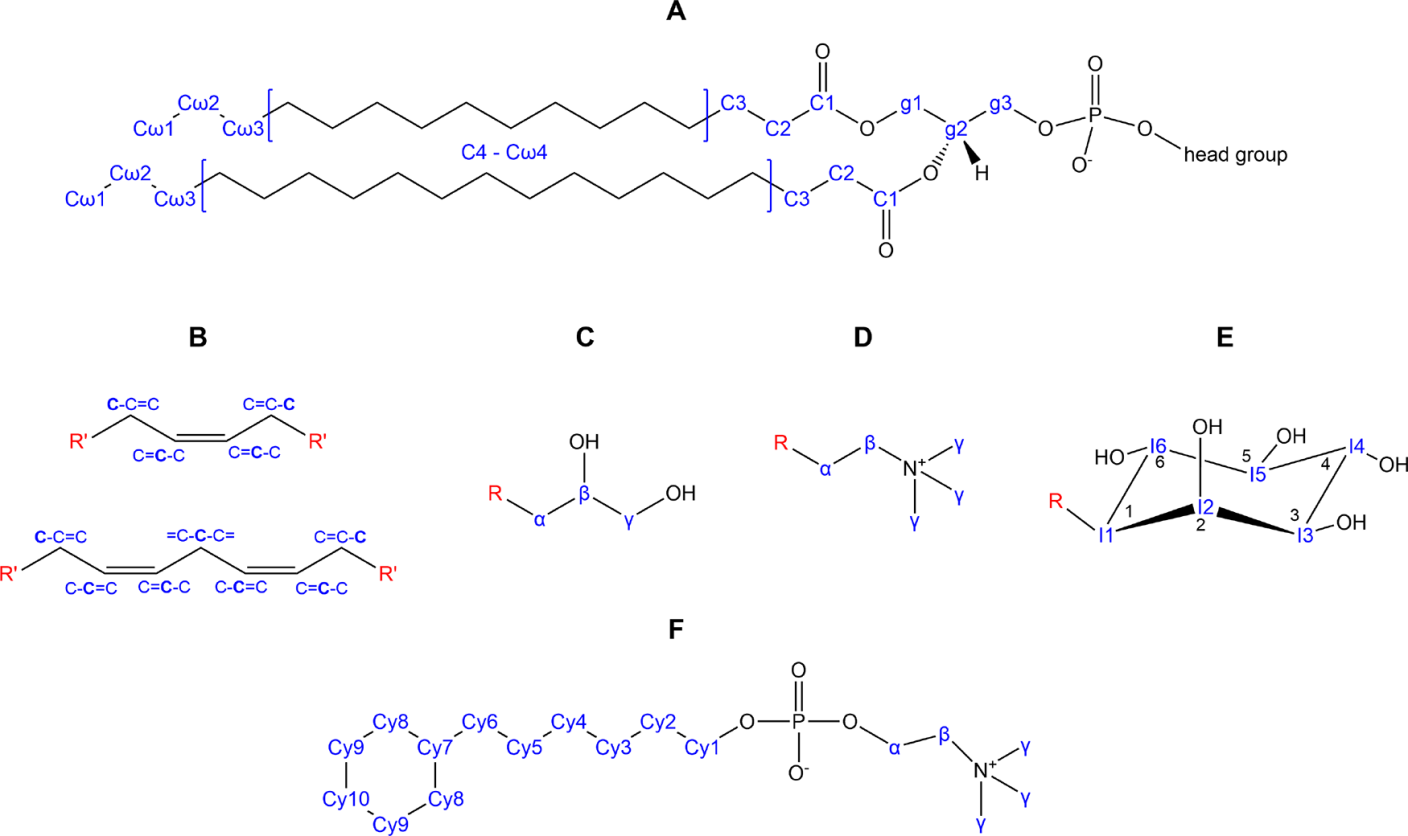
Chemical structures of the lipids used in preparing lipid/Cyclosfos-6 bicelles with the carbon nomenclature used indicated. (A) Generic phospholipid structure; (B) structures around double bonds; (C) PG headgroup; (D) PC headgroup; (E) inositol headgroup; (F) Cyclofos-6 detergent. Specific carbon nomenclature is indicated in blue.

In this work, we have developed novel, low-concentration bicelles for studying the membrane interactions of membrane-associated glycosyltransferases, such as PimA. The substrate lipid for PimA is phosphatidylinositol (PI), and it has been proposed that membrane interaction is mediated via electrostatic interactions, i.e., negatively charged lipids like phosphatidylglycerol (PG) play a key role in binding PimA to the membrane.^27,28^ To mimic various physicochemical aspects of the lipid composition of mycobacterial membranes, we have developed bicelles using Cyclofos-6 as the detergent and different combinations of PI, PG, and PC as lipids (Figure 1). These lipids were chosen since PI is the lipid substrate for PimA and also a model for lipids with an inositol headgroup, and PG has a negative charge that has been demonstrated to be important for bilayer binding. In this way, we obtained low-concentration bicelles that have the charge characteristics for studying both the mycobacterial GT PimA as well as more generic enzymes in Gram-negative bacteria, such as *E. coli* WaaG. By using NMR spectroscopy, we demonstrate that bicelles are formed, and we characterize the dynamic properties of the lipids in the bicelles using spin relaxation experiments in combination with an extended model-free approach. Finally, we show that we can use these bicelles to investigate membrane interactions of PimA.

## Materials and Methods

### Materials

6-Cyclohexyl-1-hexylphosphocholine (Cyclofos-6) was used as the detergent in the preparation of the bicelles and was purchased as powder from Anatrace Products (Maumee, OH, USA). The unlabeled lipids used were 1-palmitoyl-2-oleoyl-*sn*-glycero-3-phosphocholine (POPC), 1-palmitoyl-2-oleoyl-glycero-3-phosphatidylglycerol (POPG), and/or soy phosphatidylinositol (PI), all purchased as powder from Avanti Polar Lipids (Alabaster, AL, USA). According to the manufacturer, the fatty acid composition of the soy PI was mainly 16:0 (palmitoyl) and 18:2 (linoleoyl), with some variation in the linoleoyl chain. All lipids used in the present study have gel–liquid crystalline phase transition temperatures well below 298 K used for all measurements (271 K for glycerolipids with 16:0–18:1 chains, and well below that for 16:0–18:2 chains). All lipids were used without further purification.

### Preparation of Bicelles

Bicelle samples of *q* = 0.3 (where the molar ratio *q* = [lipid]/[detergent]) were constructed based on a method detailed in ref (26). A 10× stock ([lipid] = 81.3 mM, [detergent] = 271 mM) of each type of bicelle was made and diluted prior to use. Lipids were used directly in powder form. Cyclofos-6 was dissolved in 50 mM Tris (pH 7.5) and 150 mM NaCl buffer, and this solution was added to the lipid powder(s) in the desired proportions and vortexed to mix, resulting in a turbid slurry. This slurry was subjected to five cycles of flash-freezing in liquid N_2_, thawing in a ∼60 °C water bath, and gentle agitation, after which a clear, non-viscous solution was obtained. Five hundred microliter NMR samples with 10% D_2_O were made with a 10× dilution of the stock to obtain [lipid] = 8.1 mM and [detergent] = 27.1 mM in the following combinations and ratios of lipids: (i) 100% PI, (ii) POPC:PI [70:30], (iii) POPC:POPG [60:40], and (iv) 100% POPC. The buffer was 50 mM Tris with 150 mM NaCl at pH 7.5. D_2_O (10%) was added for frequency locking.

### Preparation of Spin-Labeled PimA^K81C^

PimA^K81C^ was produced by a QuikChange-like procedure using pET29a-*pimA* as DNA template. PimA^K81C^ was expressed and purified as described previously.^28,29^ Briefly, PimA^K81C^, containing a C-terminal, non-cleavable hexahistidine tag, was expressed in BL21(DE3) cells supplemented with appropriate antibiotics for ∼16 h at 20 °C. Cells were resuspended in 50 mM Tris–HCl (pH 7.5), 500 mM NaCl, and 15 mM imidazole and lysed by sonication, and the lysate was passed onto a HisTrap column. Protein was eluted using a linear gradient from 0 to 100% of 50 mM Tris–HCl (pH 7.5), 500 mM NaCl, and 500 mM imidazole. Next, the sample was concentrated and passed onto a Superdex 200 column for size-exclusion chromatography. To spin-label the solvent-exposed Cys residue, the procedure previously described was used.^30^ In brief, 1 mM TCEP was added to the protein solution, which was then incubated for 30 min to reduce disulfide bonds. TCEP was removed using a PD-10 desalting column. MTSL was added in 10 times molar excess of the protein, and the solution was incubated overnight at 4 °C under gentle agitation. Excess MTSL was removed by passing the sample through a PD-10 desalting column and eluted with 50 mM Tris–HCl (pH 7.5) and 150 mM NaCl buffer. Samples used for NMR experiments contained 130 μM of protein and 10% D_2_O for frequency locking.

### NMR Chemical Shift Assignment

1D ^1^H and natural abundance ^13^C spectra of bicelles were recorded on a 600 MHz Bruker Avance spectrometer, and peaks were assigned by comparing with previous assignments for lipids^22^ and the Spectral Database for Organic Compounds (SDBS).^31^ Chemical shifts for the PI inositol headgroup and acyl chains were obtained from the “D-myo-inositol-1-phosphate” entry (Compound ID E6lRHezBUnH) in SDBS and from previously published assignments. Chemical shifts for the choline group of POPC and Cyclofos-6 were taken from previous assignments,^22^ while the remaining shifts for Cyclofos-6 were assigned using SDBS.

### Translational Diffusion Experiments

Translational diffusion coefficients (*D*_t_) of bicelles were measured using a Bruker Avance spectrometer operating at 600 MHz equipped with a triple resonance ^1^H/^13^C/^15^N room-temperature probe-head using a pulsed field gradient (PFG) pulse sequence with a fixed diffusion time and a pulsed field gradient increasing linearly over 16 or 32 steps. All diffusion experiments were performed at 298 K. For all bicelle diffusion coefficient measurements, the diffusion time was set to 200 ms and the corresponding gradient pulse length was set so that the residual signal intensity at the strongest gradient was 5% of the initial intensity. These parameters were kept constant for each experiment. For water diffusion coefficient measurements, the diffusion time was set to 70 ms and the gradient pulse length was set as above. The pulse gradient field was linearly increased from 1 to 95% over 16 or 32 steps. The *D*_t_ was determined by fitting the signal intensity versus pulse gradient field strength using a modified version of the Stejskal–Tanner equation, which takes into account possible field inhomogeneities.^32,33^ The *D*_t_ was then corrected for sample-specific viscosity using the ratio between the *D*_t_ of water in the samples and a standard value of *D*_t_ = 2.3 × 10^–9^ m^2^ s^-1^.^34^

The size of a diffusing particle can be estimated from the diffusion coefficient, *D*_t_, using the Stokes–Einstein equation^35^
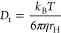
1where *k*_B_ is the Boltzmann constant, *T* is the temperature, η is the viscosity, and *r*_H_ is the radius of the sphere (the “hydrodynamic radius”). For non-spherical particles, differences between *r*_H_ and the particle’s actual dimensions arise, but the hydrodynamic radius still remains a good size estimate for near-spherical objects like small isotropic bicelles.

### ^13^C Spin Relaxation

Relaxation experiments were carried out using 500, 600, and 700 MHz Bruker Avance spectrometers operating at 125, 151, and 176 MHz ^13^C frequencies, respectively. The 500 and 700 MHz spectrometers were equipped with a cryogenic probe, and the 600 MHz spectrometer with a room temperature triple resonance probe-head. All experiments were performed at 298 K. Spectra were processed using Bruker TopSpin Software. *R*_1_ and NOE relaxation parameters were measured using natural abundance ^13^C samples. *R*_1_ measurements were conducted using an inversion recovery pulse sequence with 10 relaxation delays in the range of 0.05–6.4 s. NOE factors were measured by taking the intensity ratio between spectra acquired with and without a proton preacquisition saturation period of 25 s. An extension of the model-free analysis^36,37^ was performed to fit order parameters and correlation times. In this model, three motions are considered to contribute to spin relaxation in the bicelles: the overall bicelle reorientation, the overall lipid reorientation, characterized by *S*^2^_lip_ and τ_lip_, and local motion of individual ^13^C–^1^H bond vectors, characterized by *S*^2^_loc_ and τ_loc._^38,39,13^ The overall motion of the entire bicelle has previously been shown to be too slow to affect R_1_ and heteronuclear NOE, even for small isotropic bicelles.^39,13^ The extended spectral density function can then be written as:
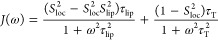
2where τ_T_ = τ_lip_τ_loc_ / (τ_lip_ + τ_loc_). This is used to calculate *R*_1_ and NOE as follows:
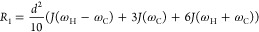

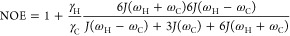
3where *d* = – μ_0_*h*γ_C_γ_H_/(8π^2^*r*^3^) and ω_C_ and ω_H_ are the Larmor frequencies of the respective nuclei, γ_C_ and γ_H_ are the gyromagnetic ratios of ^13^C and ^1^H, and *r* is the length of the ^13^C–^1^H bond vector. *R*_1_ and NOE were fitted for each measured site to give the corresponding local motional parameters, *S*^2^_loc_ and τ_loc_.

### PRE Experiments

Paramagnetic relaxation enhancement (PRE) measurements^40,41^ were carried out using a Bruker Avance spectrometer operating at 700 MHz. Measurements were carried out for MTSL-labeled PimA^K81C^ (PimA^K81C-MTSL^) in bicelles containing POPC/PI to observe effects on lipid ^13^C relaxation in the presence of the spin-labeled protein. PREs were calculated by subtracting *R*_1_ values of samples with and without the paramagnetic label following a protocol used earlier.^42^

### Dynamic Light Scattering

Dynamic light scattering (DLS) measurements were performed using a Zetasizer Nano ZS instrument (Malvern Instruments, Worcestershire, UK) equipped with a 633 nm laser with temperature set to 298 K. The freshly prepared, clear and non-viscous micelle and bicelle samples were measured in UV-transparent disposable cuvettes of 1 cm path length. A 1 min temperature equilibration delay was included prior to each experiment that consisted of at least 12 measurements of 30 s that were averaged. The scattering data was processed in Zetasizer Software v8.02 and converted to size distributions using standard sample refractive indices of 1.33 and viscosity of 0.897 cP at 298 K.

## Results and Discussion

### Bicelle Size

Translational diffusion was measured by NMR to establish whether the lipids used here and the detergent Cyclofos-6 formed bicelle-like structures. Diffusion coefficients for bicelles composed of PI, PI/POPC (30 mol %/70 mol %), POPG/POPC (40 mol %/60 mol %), and POPC are shown in Table 1.

**Table 1:** Normalized^a^ Translational Diffusion Coefficients and Hydrodynamic Radii for Lipids in *q* = 0.3 Bicelles

samples	lipid *D*_t_ (10^–11^ m^2^ s^–1^)	*R*_H_ (nm)^b^
POPC/Cyclofos-6	7.0 ± 0.03	3.5 ± 0.02
PI in Soy PI/Cyclofos-6	10.4 ± 0.3	2.4 ± 0.1
Cyclofos-6 in Soy PI/Cyclofos-6	17.0 ± 1.6	
(30% Soy PI +70% POPC)/Cyclofos-6	9.5 ± 1.5	2.6 ± 0.4
(40% POPG +60% POPC)/Cyclofos-6	5.7 ± 0.11	4.3 ± 0.1

a Normalized according to the diffusion of H_2_O.

b Based on the diffusion coefficients of the lipids.

**Figure 2:**
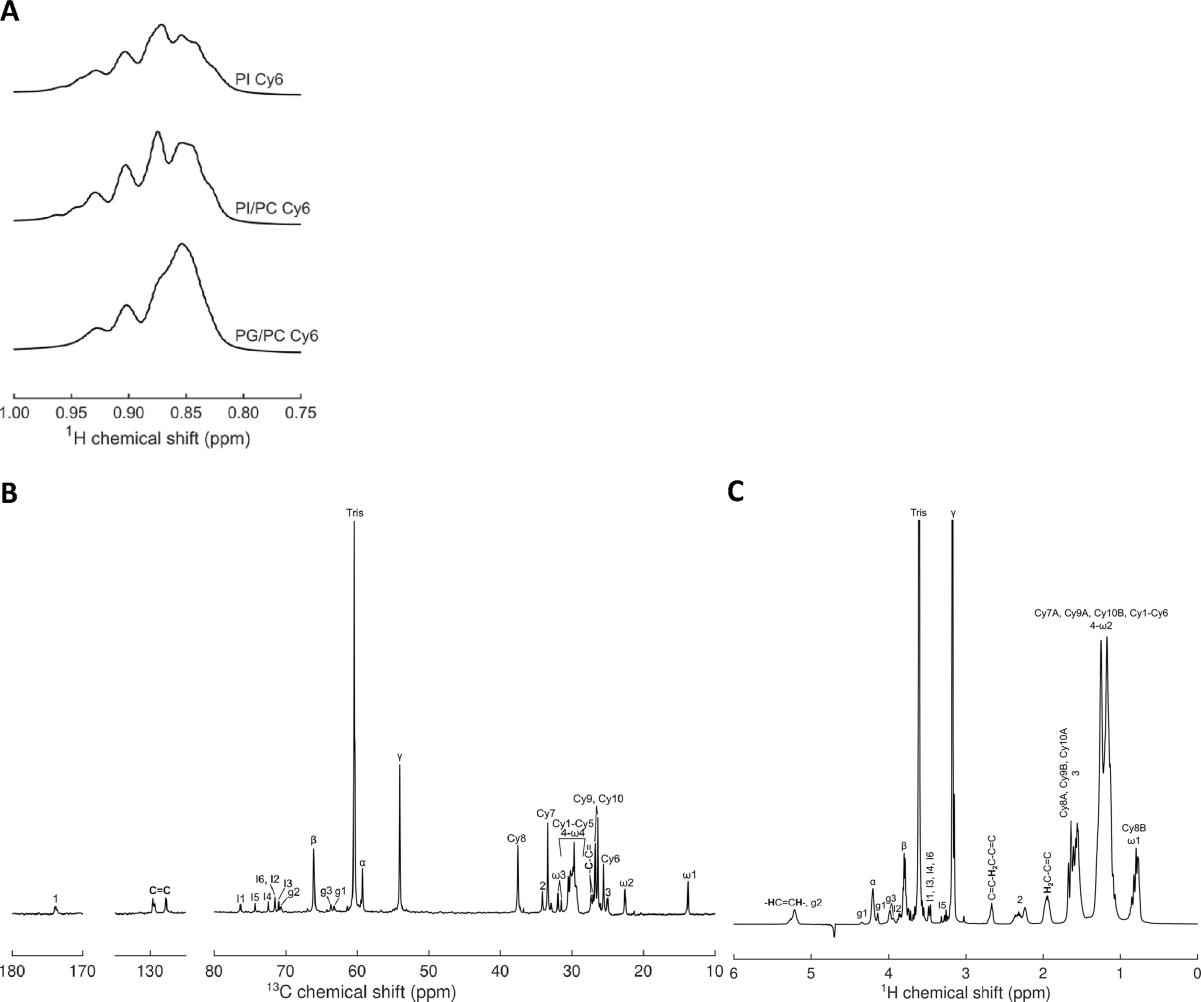
^1^H and ^13^C spectra for bicelles. (A) Methyl region of the ^1^H NMR spectra for the different bicelles recorded at 298 K. (B) ^13^C spectrum and (C) the ^1^H spectrum of the POPC/PI-Cyclofos-6 bicelle mixture with assignments for the resonances. The spectra were recorded at 298 K. The atom nomenclature for the assigned peaks is as in Figure 1.

Signals that stem only from lipids without any overlap with Cyclofos-6 detergent signals were used to calculate the diffusion coefficients (for ^1^H NMR spectra of the bicelles, see Figure S1, Supporting Information). These were signals corresponding to protons bound to g2 and g3 carbons in the glycerol part of the lipids, as well as the C2 carbon in the acyl chains. In the PI/POPC bicelles, it was possible to measure diffusion for the non-overlapping PI signal corresponding to the CH_2_ group between the two double bonds in the C18:2 acyl chain that is only present in PI. The diffusion coefficient (10.3 m^2^ s^–1^) was within the error limits of the average lipid diffusion, demonstrating that all lipids participate in the same type of assemblies. The diffusion coefficients were used to estimate hydrodynamic radii for the particles using eq 1. This simplified way of relating diffusion rates with particle size provides a convenient way of comparing diffusion factors for the different bicelle mixtures. The data shows that bicelles containing PI are significantly smaller (*R*_H_ = 2.3–2.5 nm) than bicelles containing POPC (*R*_H_ = 3.5 nm) or POPC/POPG (*R*_H_ = 4.3 nm). This is qualitatively supported by inspection of the ^1^H NMR spectra that demonstrates that the larger POPC/POPG bicelles have broader line-widths (Figure 2a). For full ^1^H spectra, see Figure S1. In all cases, the lipids have phase transitions temperatures that are well below 298 K used for the measurements, i.e., lipids are in a liquid crystalline phase under our experimental conditions. This is also supported by relatively sharp signals in both ^13^C and ^1^H NMR spectra (Figure 2B,C).^19^

Contrary to this, the diffusion coefficient for the detergent, which only had non-overlapping signals in pure PI bicelles (γ and δ ^1^Hs in the cyclosfos-6 PC headgroup), was found to be larger than for the PI lipids, 17.0 × 10^–11^ m^2^ s^–1^ vs 10.4 × 10^–11^ m^2^ s^–1^, indicating that a portion of the detergent is either found as free monomers or micellar aggregates in solution. The presence of free detergent has previously been observed in the original work using Cyclofos-6 by Lu et al.^26^ as well as in other bicelle mixtures using other detergents.

**Figure 3:**
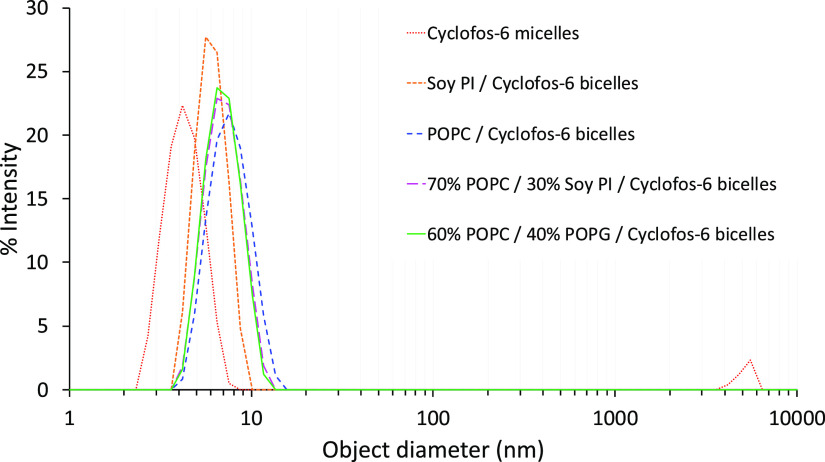
Size of bicelles and Cyclofos-6 micelles as determined by DLS.

The diffusion coefficients measured by NMR could potentially be averages of coefficients from two or several species with different sizes in solution, which are difficult to resolve using the PFG diffusion experiment. Therefore, the NMR data was complemented by DLS experiments, which are sensitive to diffusive motion on a shorter time scale than NMR and can thus resolve smaller particle size differences and also be capable of detecting objects beyond the upper size limit of solution NMR. No additional peaks corresponding to particles of different sizes than those corresponding to bicelles as measured by NMR diffusion were observed (Figure 3), suggesting that the samples consist of well-defined bicelle particles. For the Cyclofos-6 micelle sample, the presence of a small amount of a micrometer-sized object was detected but was likely due to an impurity and not related to the detergent as none of the bicelle samples displayed a corresponding population. In addition, it was seen that PI bicelles are indeed smaller than bicelles with PC, supporting the translational diffusion data and that bicelles are significantly larger than micelles formed by Cyclofos-6 alone.

In conclusion, the diffusion data demonstrates that PI lipids are incorporated into bicelles using Cyclofos-6 as the detergent. Bicelles made with PI are, however, smaller than bicelles with POPC and POPC/POPG, something that may indicate either a higher degree of mixing between the lipids and detergent or could be a result of a very different headgroup affecting bilayer properties.^43^ Importantly, however, all mixtures used here with Cyclofos-6 form one set of assemblies and stable bicelles are formed even with a 5-fold lower lipid and detergent concentration as compared to “conventional” bicelles prepared with DHPC as the detergent.

### Lipid Relaxation in Bicelles

The dynamics of lipid molecules carries information about how bilayer-like the environment is.^13,21,22^ We therefore measured ^13^C relaxation at three magnetic fields for the lipids in the three novel bicelle mixtures containing PI, PI/POPC (30 mol %/70 mol %), and POPG/POPC (40 mol %/60 mol %). The assignment of resonances in the ^13^C and ^1^H spectra of POPC/PI bicelles can be found in Figure 2B. The ^13^C spectra of all three bicelles are found in the Supporting Information (Figures S2–S4). Many of the carbon resonances overlap for acyl chains in different lipids, and therefore, only averages for all lipid chains are reported. Only data for carbons that have resonances that do not overlap with other types of carbons are included in the analyses. Some resonances are, however, clearly identifiable as belonging to certain lipid molecules, such as headgroup resonances, and double bonds in the unsaturated chains and these are reported independently.

**Figure 4:**
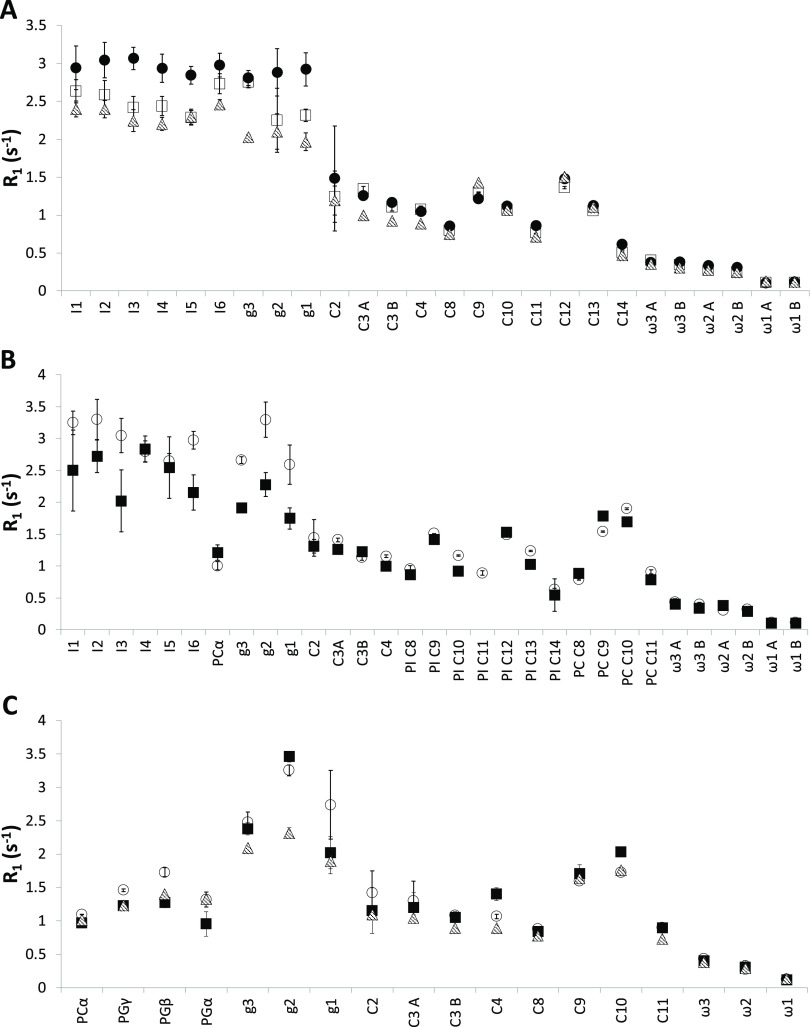
R_1_ data for *q* = 0.3 bicelles prepared with Cyclofos-6 as the detergent and PI (A), PI/POPC (30 mol %/70 mol %) (B), and POPG/POPC (40 mol %/60 mol %) (C). The data were measured at 298 K and at three magnetic field strengths: 11.75 T (filled circles), 14.1 T (open squares) and 16.45 T (hatched triangles). The values have been normalized to reflect the relaxation for one ^13^C–^1^H vector for each site. A and B refers to the same position in the two different acyl chains in the molecule. No specific assignment for the two chains was made. The carbon atom nomenclature is as in Figure 1.

**Figure 5:**
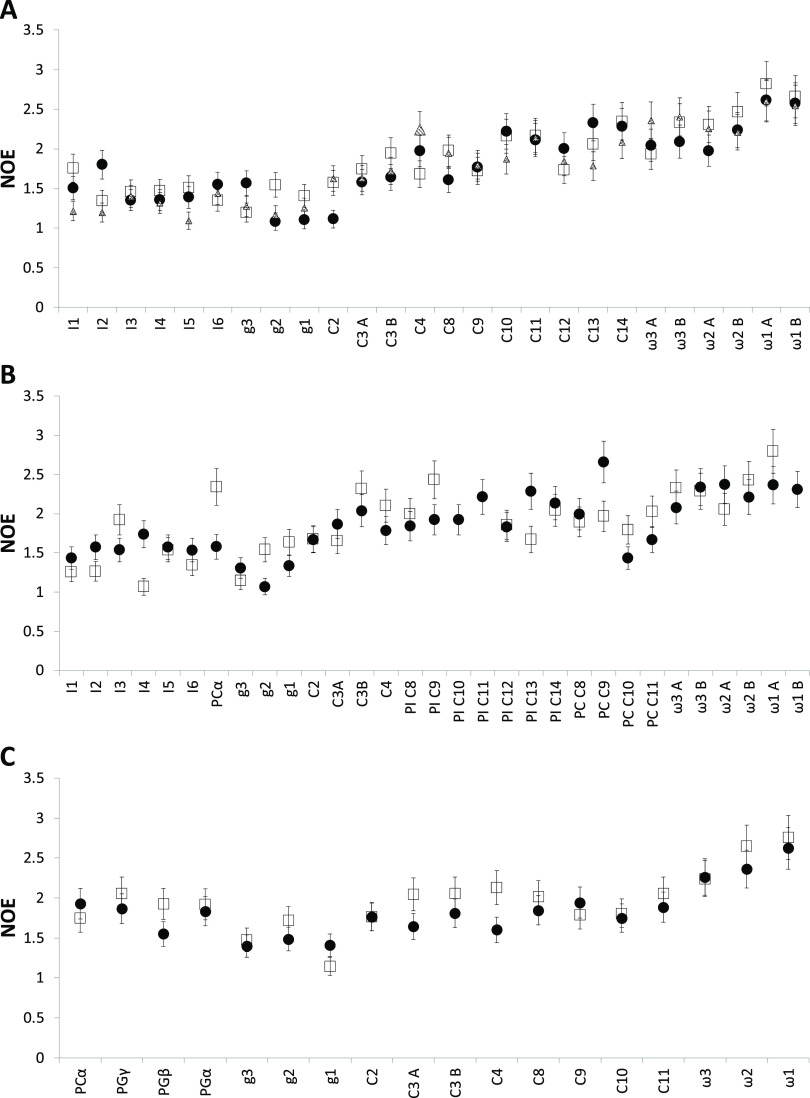
NOE data for *q* = 0.3 bicelles prepared with Cyclofos-6 as the detergent and PI (A), PI/POPC (30 mol %/70 mol %) (B), and POPG/POPC (40 mol %/60 mol %) (C). The data were measured at 298 K and at three magnetic field strengths: 11.75 T (filled circles), 14.1 T (open squares), and 16.45 T (hatched triangles). A and B refers to the same position in the two different acyl chains in the molecule. No specific assignment for the two chains was made. The carbon atom nomenclature is as in Figure 1.

*R*_1_ relaxation rates for the three bicelles are collected in Figure 4, and NOE parameters are collected in Figure 5. The PI inositol moiety (Figure 3A,B) together with the glycerol backbone in all lipids has very different *R*_1_ relaxation behavior compared to the other parts of the lipid, indicating slower internal motion for the inositol headgroup. In contrast, *R*_1_ relaxation rates for PC and PG headgroups are lower, indicating that these are more flexible than PI headgroups. In all cases where it is possible to separate resonances originating from different lipid molecule chains, there is very little difference between the different lipids, indicating that all chains have the same relaxation behavior and, therefore, the same dynamics. It is also clearly possible to discern double bonds in the acyl chains (at position C9, C10 in the palmitoyl chain in POPC, and C9, C10, and C12, C13 in the linoleoyl chain) that display higher *R*_1_ values compared to neighboring carbon atoms, again indicative of slower motion. Toward the end of the acyl chains, relaxation is very slow (on the order of 0.1 s^–1^), indicating more or less unrestricted motion. For POPC, in PI/POPC bicelles (Figure 3B) and POPG in POPG/POPC bicelles (Figure 3C), the relaxation data agree well with what has previously been determined.^13,21,22^

For ^13^C NOE values, the same trend as observed for the *R*_1_ data can be seen, although not as clearly (Figure 5). This is due to the larger errors that are associated with the determination of NOE values as compared to *R*_1_. Parts of molecules that have a higher degree of internal dynamics generally display larger NOE values, and this is also observed in the NOE data. Very little NOE is observed for the inositol headgroup in PI (Figures 4A,B), with values between a little more than 1 (no NOE) up to around 1.7. Contrary to this, the terminal carbons of the acyl chains have NOE values that are around 2.5, closer to the theoretical maximum NOE of 3.

### Lipid Dynamics in Bicelles

To obtain a better understanding of the underlying dynamics that give rise to the relaxation behavior, an extended model-free analysis of the relaxation data was performed. The analysis was performed as described in the Materials and Methods section. Fitting the data to eqs 2 and 3, keeping the parameters for the lipid reorientation fixed yielded a correlation time and an order parameter for the internal local motion of each ^13^C–^1^H bond vector.

Figure 5 shows the fitted order parameters (*S*^2^_loc_) for each site in the lipids in the three different bicelle types. The fitting reveals that as expected, the restriction in local motion follows the measured relaxation parameters closely and provides a good measure of the degree of spatial restrictions of various sites in the lipid molecules. The order parameters for the inositol moiety in bicelles with PI are relatively high, in the range of 0.6–0.9. The data clearly indicates that the ^13^C–^1^H bond vectors in the PI inositol headgroup in both PI (Figure 5A) and PI/POPC bicelles (Figure 5B) have relatively high order parameters, again demonstrating that they are rigid in bicelles. In contrast to the PI head group, the head groups of PC and PG (Figure 4C), which are both rather small, display fast dynamics that are similar to the upper part of the acyl chains. Note, however, that headgroup resonances of POPC and Cyclofos-6 overlap. Nevertheless, the order parameters for the PC headgroup agree well with what has previously been observed in bicelles composed of different lipid mixtures, including PC.^13,22,21^

The glycerol backbone of the lipids in all bicelles display very limited motion, an observation that is consistent with results for other phospholipids in bicelles using DHPC as the detergent.^13,22,21^ For the acyl chain, the degree of motional freedom increases farther out in the chain, with the exception of the unsaturations in the linoleoyl and palmitoyl chains that locally restrict dynamics.

**Figure 6:**
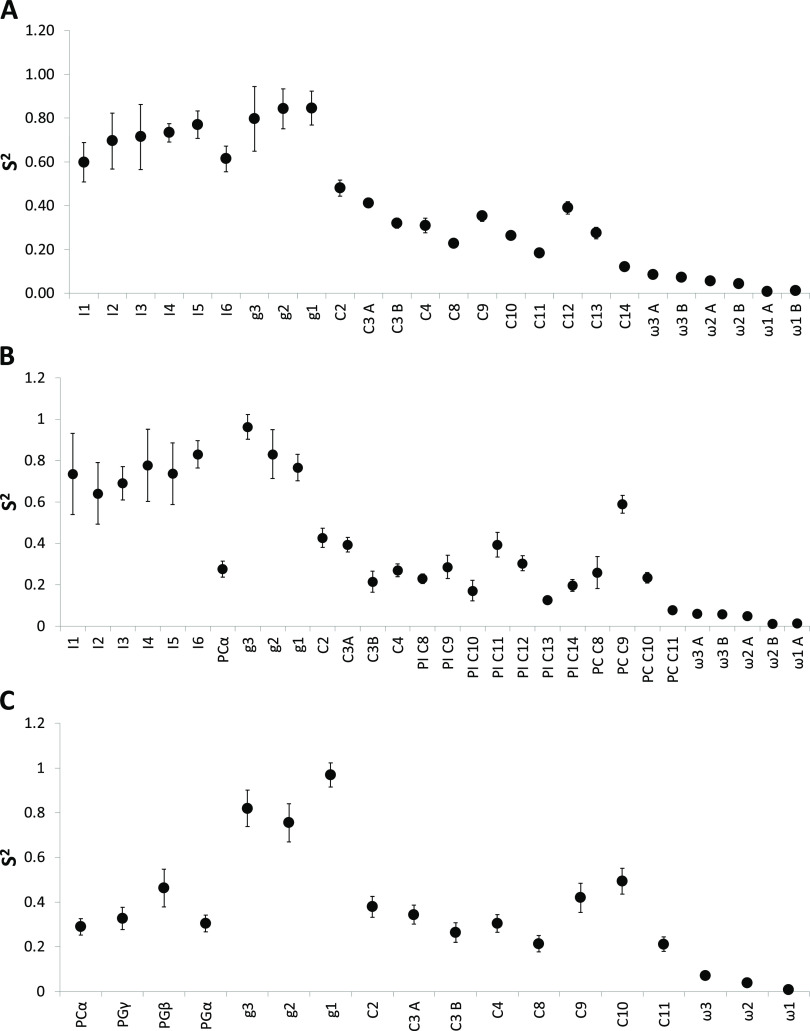
*S*^2^_loc_ from an extended model-free analysis of *R*_1_ and NOE data for *q* = 0.3 bicelles prepared with Cyclofos-6 as the detergent and (A) PI, (B) PI/POPC (30 mol %/70 mol %), and (C) POPG/POPC (40 mol %/60 mol %). The carbon atom nomenclature is as in Figure 1.

The order parameters for bicelles containing POPG/POPC are very similar to what has previously been recorded, e.g., for lipids in DMPC/DMPG/DHPC bicelles, indicating that the choice of detergent does not appear to influence the dynamics of the individual lipid molecules. It is moreover worth noticing that the much lower overall concentration of lipids and detergents used in this study does not seem to have an effect on lipid dynamics (Figure 6).

**Figure 7:**
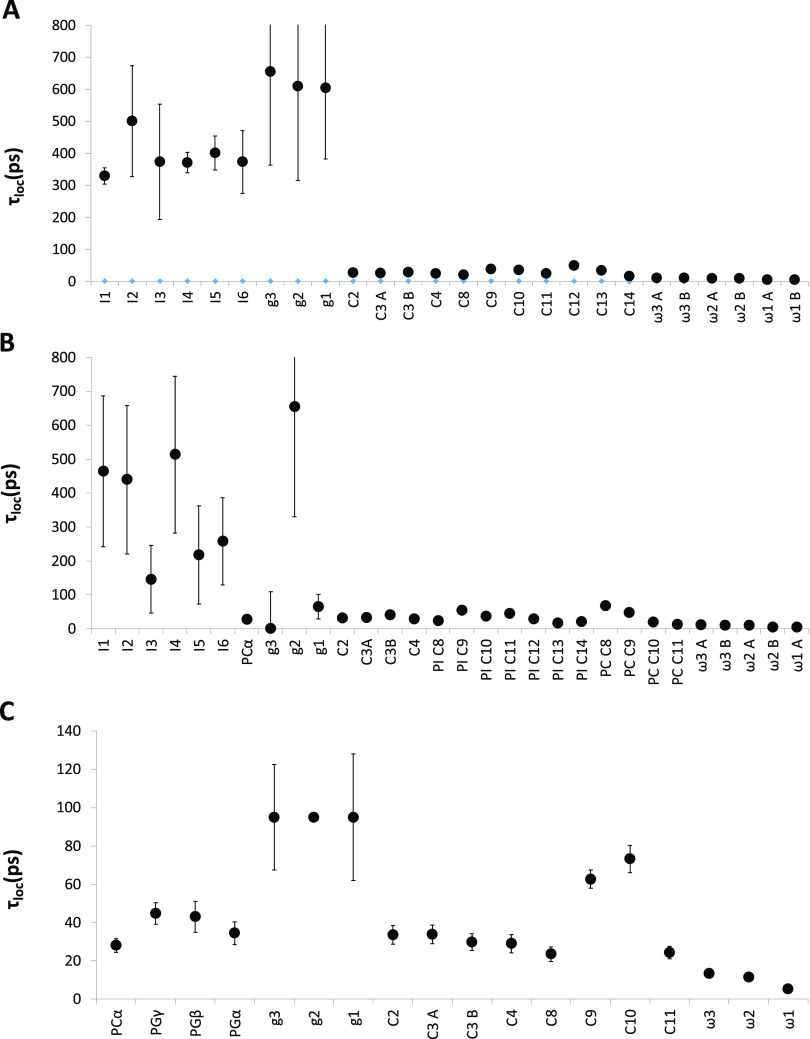
τ_loc_ from an extended model-free analysis of *R*_1_ and NOE data for *q* = 0.3 bicelles prepared with Cyclofos-6 as the detergent and (A) PI, (B) PI/POPC (30 mol %/70 mol %) and (C) POPG/POPC (40 mol %/60 mol %). The carbon atom nomenclature is as in Figure 1.

Local correlation times extracted from the fitting are collected in Figure 7. Again, the values for the three bicelle types correlate very well with the order parameters and with the initial observations made already in the relaxation data. The correlation times for the inositol headgroup carbons as well as the glycerol carbons are relatively long with values up to around 700 ps, while the correlation times for the acyl chains are very short (less than 100 ps). This indicates that the dynamics for the inositol moiety approaches the time-scale for the reorientation of the entire lipid. This has previously been observed, e.g., for galactolipids in bicelles^22^ and indicates that the two motions may be coupled. In one of the bicelles, PI/POPC, fitting for two of the correlation times for the glycerol carbons did not yield reliable values, which also indicates that the motional model used here may not be accurately analyzing motions that are not well separated in time. For all carbons in the acyl chains, irrespective of which lipid mixture, the correlation times were very short, in agreement with the order parameters and indicating that the reorientation time of the ^13^C–^1^H bond vector is very fast in the acyl chains. As for the order parameters, it is also possible to see the variation in motional freedom for the carbons participating in double bonds, although all correlation times for the lipid chains are in the same fast motional regime as compared to the inositol headgroups.

Taken together, the results indicate that the lipids behave in a similar way as previously seen for bicelles at higher concentrations and with different detergents. We also conclude that the inositol headgroup in the PI lipid is relatively rigid and that this does not seem to affect the bicelle-forming properties of this lipid. Two important conclusions can therefore be drawn from the present results. The first is that using the detergent Cyclosfos-6 to form low-concentration bicelles preserves bilayer-like lipid behavior. This is important as it has been demonstrated that the choice of detergent can indeed affect the dynamic properties of the lipid molecules. For example, CHAPS has been seen to induce more rigidity in lipid tail motion than what is observed in bicelles with DHPC as detergent.^13,19^ The second conclusion is that introducing PI into bicelles, either as the only lipid or in combination with PC, does not seem to alter the behavior of individual lipid molecules.

### PimA Binding to Bicelles

**Figure 8:**
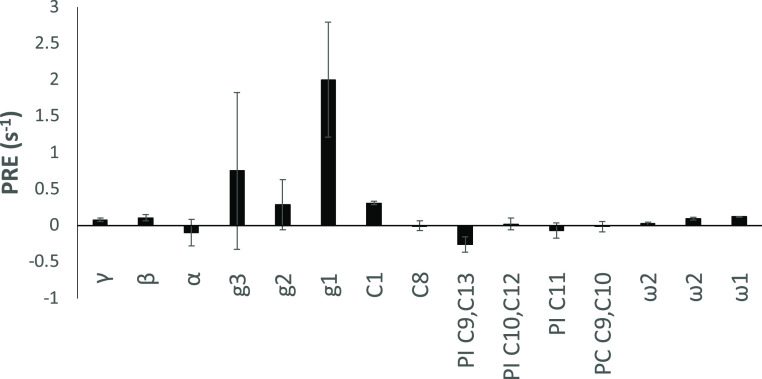
Paramagnetic relaxation enhancement (PRE) effects on carbons in PI/POPC (30 mol %/70 mol %) bicelles from PimA^K81C-MTSL^. The errors are estimated from the errors in the measured *R*_1_ values. The carbon atom nomenclature is as in Figure 1.

To test if PI-containing bicelles could be used for detecting interaction with PimA, we measured PimA induced paramagnetic relaxation enhancement on the lipids. For this, we used a variant of PimA in which the paramagnetic label MTSL had been incorporated. In this variant, Lys81 was mutated to a Cys residue (PimA^K81C^) to which the paramagnetic label MTSL was attached (PimA^K81C-MTSL^). This part of the protein, in the N-terminal domain, has been suggested to take part in membrane binding,^27,28,44^ and therefore, it would be expected that a paramagnetic probe at this site should have an effect on lipid sites in its vicinity. Experiments were performed by measuring *R*_1_ with PimA^K81C-MTSL^ and subtracting *R*_1_ values from an experiment using unlabeled PimA^K81C^ using bicelles composed of POPC/PI. No data from the inositol headgroup were included since data for these carbons had very low signal-to-noise ratios. The data is collected in Figure 8.

Although we did not quantify the amount of paramagnetic label that had been incorporated into the protein, the data demonstrates that the label is present as an effect is clearly seen. It is, however, difficult to draw any quantitative conclusions about the PRE effect. The data indicates that the region that is mostly affected by the protein in the lipid molecules clearly belongs to the glycerol moiety of the lipids. Due to complete overlap between the glycerol part in PI and POPC, no distinction between the two could be made. The only significant PREs were in fact observed for this part together with C1 in the acyl chains, while no significant PREs could be seen for the other chain carbons or for the PC headgroup. The effects are also quite small, indicating either that the interactions are weak or that the relative amount of labeled protein is still small compared to the amount of lipids. Nevertheless, we see a clear effect of labeled protein primarily on the glycerol part of the lipids. Hence, we conclude that PimA interacts only weakly with the lipids and that it has a shallow location in the bilayer as demonstrated by PREs being observed only in the glycerol region. Moreover, the region in PimA that harbors the MTSL is here observed to indeed interact with bicelles. We also attempted to determine PREs in the same way for a similarly labeled PimA using conventional bicelles with PI present and with DHPC as detergent (data not shown). These measurements did not reveal any effect of MTSL-bound PimA on the lipids. In this case, the total lipid + detergent concentration had to be kept at least 10-fold higher than when using bicelles with Cyclofos-6, and we conclude that the high concentration of lipids needed when using conventional bicelles precludes any kind of interaction to be probed by investigating lipids since the protein-to-lipid ratio becomes too low to detect any PRE effects. We conclude that it is indeed possible to use low-concentration bicelles to investigate the effect that PimA has on lipids and thereby also to probe the location of the protein in a bilayer.

## Conclusions

In this work, we have developed a new set of membrane mimetics for use in studies of glycosyltransferases that are associated with phosphatidylinositol lipids. Notably, low-concentration bicelle mixtures were prepared by using a detergent, Cyclofos-6, that has previously been demonstrated to allow for bicelles to form at lower concentrations than with, e.g., DHPC.^26^ In the present study, a >5-fold lower concentration of the Cyclosfos-6-containing bicelles as compared to conventional bicelles containing DHPC were studied. Three different mixtures were examined, two with varying amounts of PI and one in which PI was replaced by PG. All three mixtures formed well-defined particles in which the lipids display dynamics that are comparable to what has been observed for other lipids in membrane mimetics, including bicelles. The bicelles with PI were however smaller than the ones with PG, indicating that PI causes a change in the morphological properties of the bicelles. We also demonstrated that the novel low-concentration bicelles can be used to probe the location of a glycosyltransferase, PimA, with respect to the lipids in the bicelles. We foresee that this will be possible for other proteins as well and on a more detailed level.

## Supporting Information Available

^1^H NMR spectra of bicelles; ^13^C NMR spectra of bicelles (PDF)
