# Supplementary material for: Dilute Bicelles for Glycosyltransferase Studies, Novel Bicelles with Phosphatidylinositol
Source: J Phys Chem B. 2022 Jul 26;126(30):5655–66. doi: 10.1021/acs.jpcb.2c02327 (PMC9358657; doi:10.1021/acs.jpcb.2c02327)
Supplement: Supplementary file 1 — jp2c02327_si_001.pdf [file jp2c02327_si_001.pdf]

Dilute bicelles for glycosyltransferase studies, novel bicelles with phosphatidylinositol

Supporting information

*Joan Patrick[1], Mikel García Alija[1,2], Jobst Liebau[1,3], Pontus Pettersson[1], Ane Metola[1] and Lena Mäler[1]**

[1] Department of Biochemistry and Biophysics, Stockholm University, SE-106 91 Stockholm, Sweden

[2] Present address: Structural Glycobiology Laboratory, Biocruces Health Research Institute, Barakaldo, Bizkaia, 48903, Spain

[3] Present address: Institute of Biophysics and Physical Biochemistry, University of Regensburg, Universitätsstrasse 31, 93053 Regensburg, Germany

*Corresponding author: Lena Mäler, email: lena.maler@dbb.su.se

SUPPORTING FIGURES

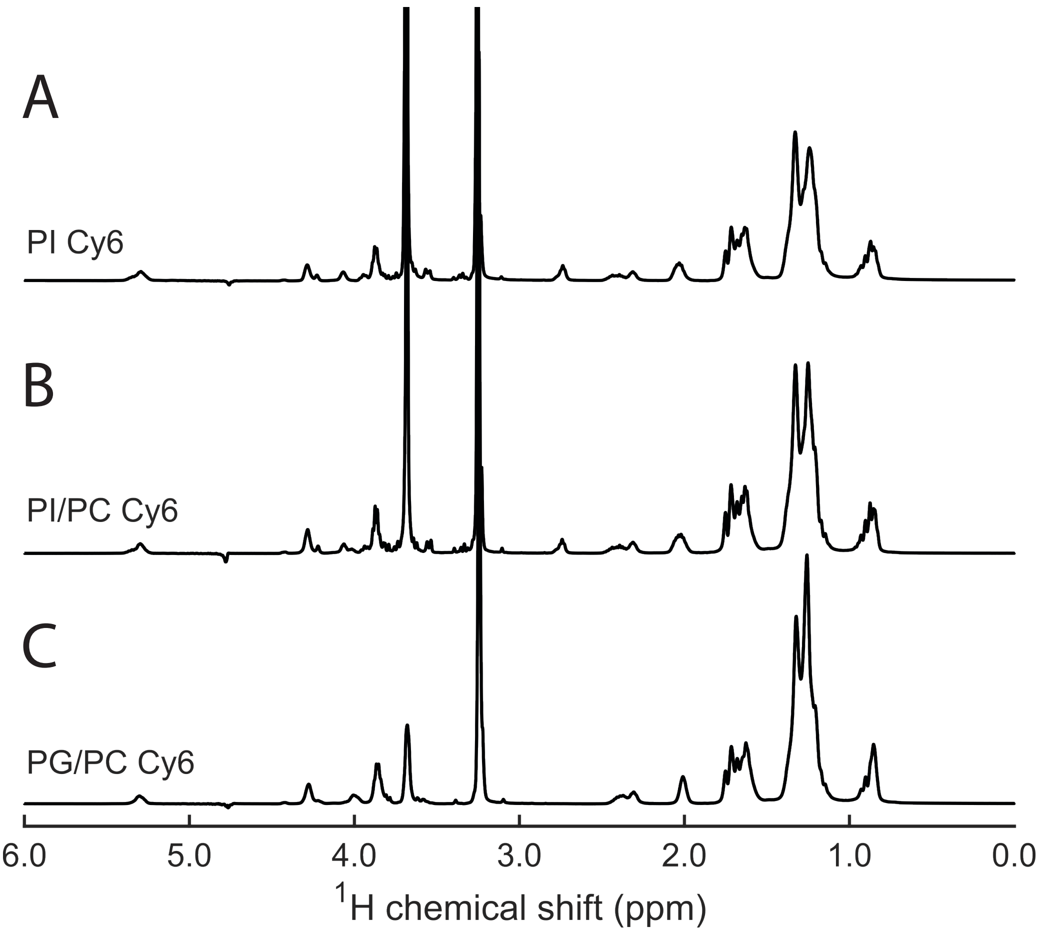

Figure S1. ^{1}H NMR spectra of the bicelles. A) Soy PI / Cyclofos-6; B) 30% Soy PI / 70% POPC / Cyclofos-6; C) 40%POPG / 60% POPC /Cyclofos-6.

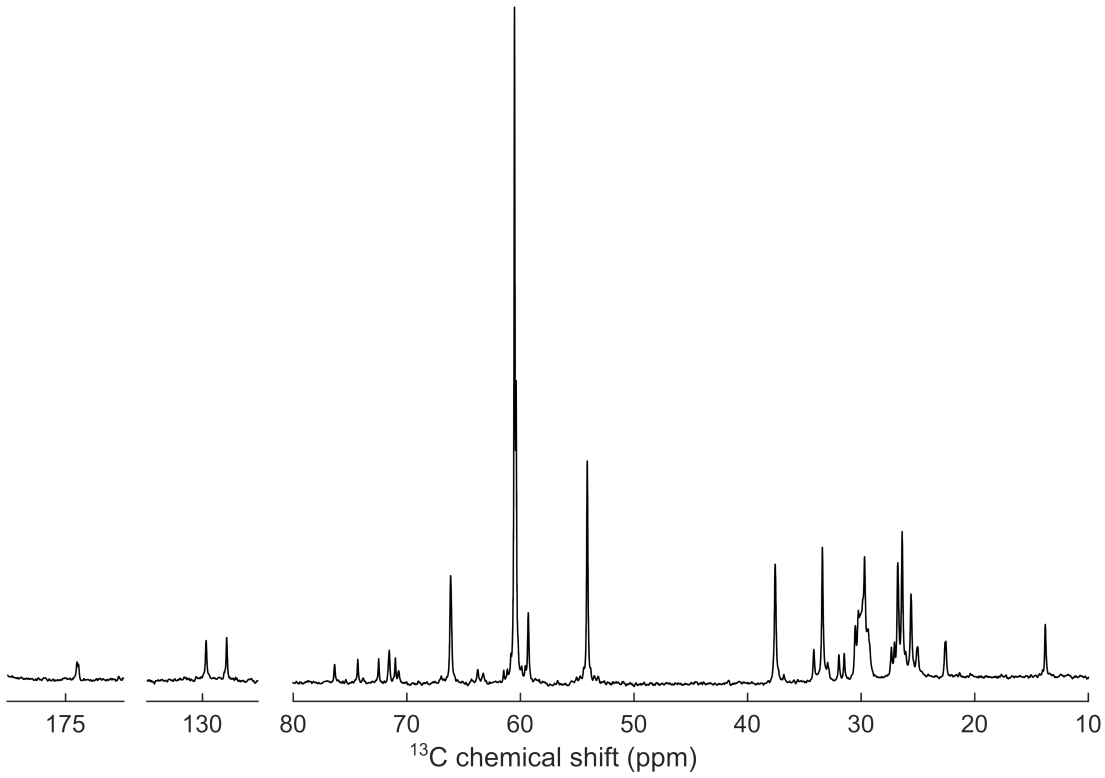

Figure S2. ^{13}C spectrum of Soy PI / Cyclofos-6 bicelles. The spectrum was recorded at 298 K.

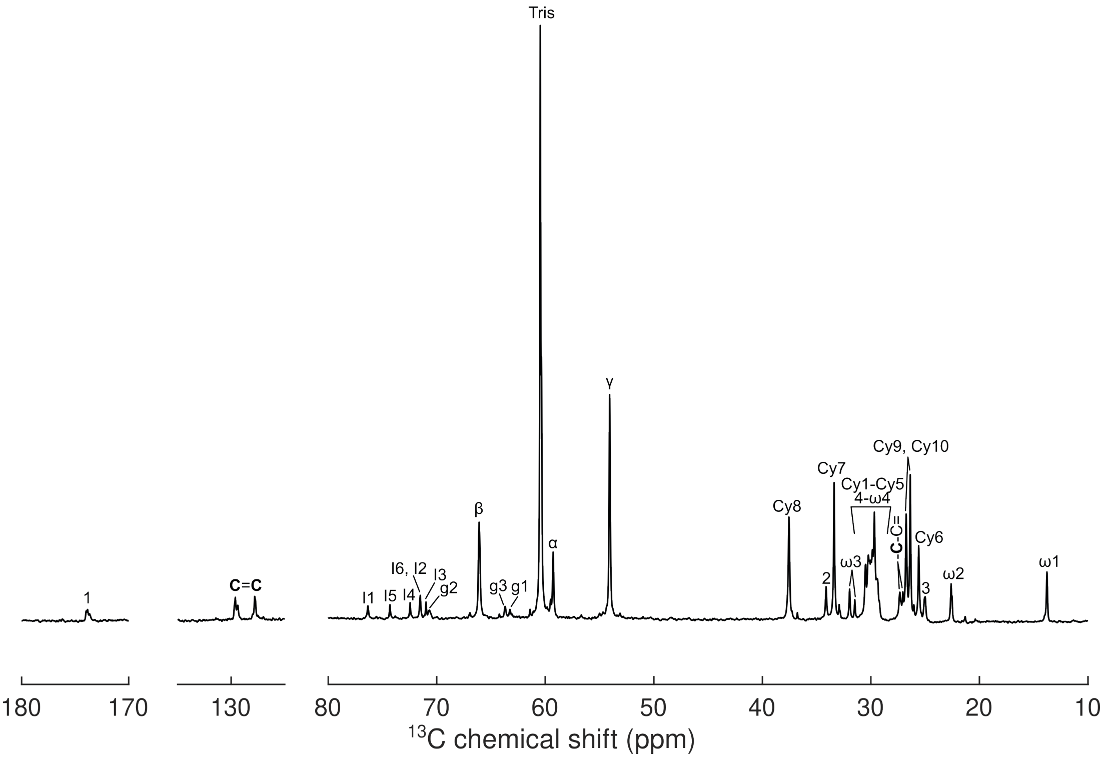

Figure S3. ^{13}C spectrum of 30 % Soy PI / 70% POPC / Cyclofos-6 bicelles with indicated assignments. The spectrum was recorded at 298 K.

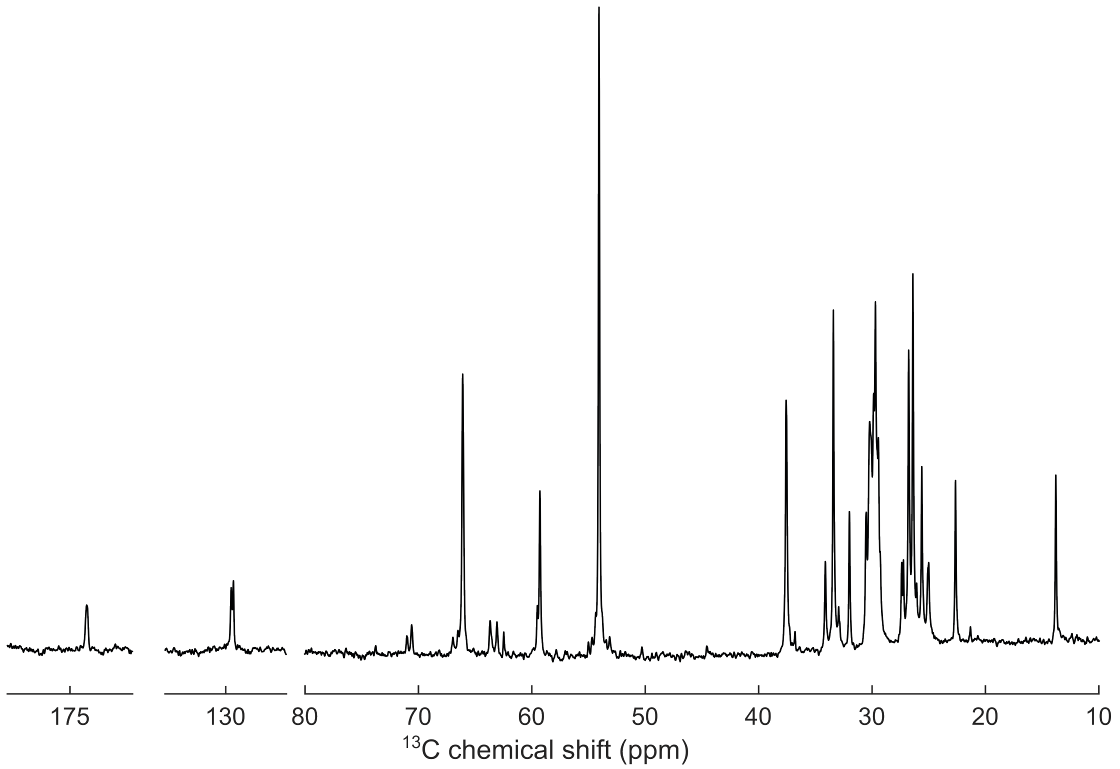

Figure S4. ^{13}C spectrum of 40% POPG / 60% POPC / Cyclofos-6 bicelles. The spectra were recorded at 298 K.
